# Successful Repetition of Wurtzer’s Method for the Radical Cure of Inguinal Hernia, in a Case of 20 Years Standing

**Published:** 1859-09

**Authors:** W. C. Smith

**Affiliations:** Grantville, Ga.


### ARTICLE II.

*Successful Repetition of Wurtzer's Method for the Radical Cure of Inguinal Hernia, in a case of 20 years standing.*—By W. C. SMITH, M. D., of Grantville, Ga.

Having witnessed the successful performance of this operation by Dr. Choppin, of New Orleans, while on a visit to that city last winter, I was so favorably impressed that I resolved to try it in a case which at that time I had in charge.

The patient in this case was a negro man, aet. 38, who was ruptured when 18 years old, by a heavy strain while lifting. The ring was large, and protrusion considerable, so that it was difficult to retain the protruded bowel with a well-adjusted truss. I requested Dr. Peddy, of Franklin, who was a member of the graduating class in the New Orleans School of Medicine, and who had frequently assisted in these operations, to examine him, which he did, and decided that the case was rather an unfavorable one, yet perhaps we might succeed in making a partial, if not a final cure. Consequently, on the 4th of July, after having put the patient under the influence of [illegible]'oroform, we proceeded to adjust the instruments according to the usually directed plan, without pain, and retained them in place by bandages, we afterwards gave him twenty drops of laudanum, ordered light and nourishing diet, and to be kept in a recumbent posture. No febrile excitement of consequence ensued. I opened his bowels on the third day with salts, and removed the instruments on the ninth day after the operation; permitted him to walk about the room for a few days, and then applied a truss and sent him out. On the 20th day, I examined him very carefully and found no opening, the parts having all healed. Then sent him out to work on the railroad, where he has been lifting and straining ever since, without any tendency to a recurrence of the affection. He still wears the truss, and will for a few weeks longer.

I am not disposed, at present, to offer any comment on the merits of this method of operating, yet I must confess that I believe the time not far distant when it will receive the serious attention of many who now are disposed to pass it lightly by.

